# Home Hospice Versus Other Places of Death and Caregiver Stresses

**DOI:** 10.1093/geroni/igaa057.1349

**Published:** 2020-12-16

**Authors:** Elizabeth Luth, Holly Prigerson

**Affiliations:** Weill Cornell Medical College, Larchmont, New York, United States

## Abstract

As of 2017, more individuals in the U.S. die at home than in any other location. Hospice care was designed to provide support for people who are dying and their families. However, dying persons may have rapidly emerging needs that home hospice does not immediately meet, thereby, exposing family members to be “first responders.” Thus, home death may result in distress and burden for dying individuals’ family members, even when hospice is involved. This study uses multivariable regression analysis to explore the relationship between place of death and stressful end-of-life experiences in a sample of 185 patients with advanced cancer. We also analyze which end-of-life experiences are associated with death location. Compared to home hospice death, we found dying in a hospital was associated with fewer caregiver exposures to, and reports of fearfulness and helplessness in response to, stressful end-of-life events. Compared to home hospice death, hospital death was associated with decreased frequency of choking, falls, confusion/delirium, and feeling the patient has had enough. It was also associated with less fearfulness about choking and falls and less hopelessness about falls, the patient having enough, and thinking the patient was dead. Our results suggest home death with hospice care, often involves undesirable experiences that result in more caregiver fear and helplessness than dying in a hospital without hospice care. Research is needed to understand how to best support family members through stressful end-of-life experiences even when supported by home hospice services.

